# Maternal Multiple Sclerosis and Offspring’s Cognitive and Behavioral Development: What Do We Know until Now?

**DOI:** 10.3390/children9111716

**Published:** 2022-11-09

**Authors:** Martina Siracusano, Elisa Carloni, Assia Riccioni, Marialaura Ferrara, Chiara Scoppola, Lucrezia Arturi, Cinzia Niolu, Girolama Alessandra Marfia, Luigi Mazzone

**Affiliations:** 1 Department of Biomedicine and Prevention, University of Rome Tor Vergata, Via Montpellier 1, 00133 Rome, Italy; 2 Child Neurology and Psychiatry Unit, Department of Neurosciences, Policlinico Tor Vergata Foundation Hospital, Viale Oxford 81, 00133 Rome, Italy; 3 Systems Medicine Department, University of Rome Tor Vergata, Montpellier Street 1, 00133 Rome, Italy; 4 Psychiatry and Clinical Psychology Unit, Department of Neurosciences, Policlinico Tor Vergata Foundation Hospital, Viale Oxford 81, 00133 Rome, Italy; 5 MS Clinical and Research Unit, Department of Systems Medicine, Tor Vergata University, 00133 Rome, Italy

**Keywords:** Multiple Sclerosis, offspring, children, development, cognitive, behavior, maternal condition

## Abstract

Multiple Sclerosis (MS) is a chronic pathological condition representing one of the main causes of neurological disability in the female young population. MS, as an immune disorder, could impact fetus development, and, considering the need for and the possibility of pharmacological treatment during pregnancy, the possible influence of medication on developmental trajectories represents a topic of great interest. We provide an overview of the available literature on the influence of maternal Multiple Sclerosis on offspring cognitive and behavioral development. A study was conducted on Pubmed, Medline and Google Scholar, considering empirical studies and reviews exclusively in the English language. Maternal MS appears not to be associated with emotional and behavioral problems, as evaluated through retrospective studies. However, a specific cognitive and behavioral phenotype, through the administration of standardized instruments, has not been delineated yet. Available studies on the topic are characterized by poor methodology and do not lead to conclusions. This overview highlights implications for further longitudinal studies which should delineate offspring developmental trajectories, taking into consideration maternal confounding factors and the exposure to pharmacological treatment in pregnancy.

## 1. Introduction

The possible impact of disorders affecting women during pregnancy on their offspring development represents a topic of growing interest from both clinical and research points of view.

A maternal pathological condition may have consequences on the newborn through several mechanisms, with heightened inflammation directly associated with maternal chronic illness having a major role in fetus development [1,2].

As a matter of fact, maternal high inflammatory levels (especially IL-6) during pregnancy have been linked with several maternal prenatal physical (infections, chronic immune diseases, obesity) and mental conditions (stress, depression, anxiety) and have been associated with adverse biological and behavioral outcomes in offspring [3,4,5,6,7,8,9,10]. However, the extent to which maternal inflammation during critical phases of prenatal life is directly linked to newborn development—and neurodevelopmental disorders’ etiopathogenesis—is widely unknown, leaving open questions.

Moreover, the emotional impact that a parental chronic condition may have on the whole family’s wellbeing, including children, can be remarkable (need for frequent hospitalization, which implies the absence of a caregiver; deterioration of a parent’s physical appearance; lack of a parent’s autonomy), leading to offspring distress and possible behavioral dysregulation [11,12,13,14].

In this context, several maternal chronic conditions have been identified as possible risk factors for the occurrence of atypical developmental trajectories and neurodevelopmental disorders in offspring, such as obesity, gestational diabetes mellitus, autoimmune diseases, hypertensive disorders, infections, mood disorders, anxiety and neurological conditions [15,16,17,18,19,20].

Among these pathological conditions affecting women, there is Multiple Sclerosis (MS), a neurologic disorder of the central nervous system representing one of the main causes of neurological disability in the female young population [21].

MS is an immune-mediated disease involving both the innate and the adaptive immune systems. A key role is played by T and B lymphocytes, which autoreact against specific antigens expressed in the central nervous system. Macrophages and microglia are also implicated, generating an inflammatory path which leads to neurodegeneration, with axonal and neuronal loss and demyelination [21,22,23]. The clinical manifestation of MS is widely variable, with different possible expressions (relapsing-remitting, primary progressive or secondary progressive). Most common symptoms related to the affected area of the central nervous system include vision deficits, motor and equilibrium impairment, dysphagia, speech difficulties, fatigue, seizures, cognitive impairment and psychiatric comorbidities such as depression, leading to significant disability [22,23,24,25].

Disease-modifying treatments (DMTs) represent the first-line therapy for women with MS. Several studies evaluated the effect of DMTs on the fetus exposed during pregnancy. For ethical reasons, most of them are retrospective or based on large databases; in other cases, studies evaluate women who accidentally had therapy in the first trimester while they were unaware of the pregnancy.

Here, we summarize the main knowledge on the use of the most common DMTs during pregnancy: interferons (IFN), glatiramer acetate (GA), dimethyl fumarate (DMF), fingolimod, teriflunomide, natalizumab and ocrelizumab (Table 1).

Regarding IFN, extensive independent and branded studies on wide registrational databases found that exposure before or during pregnancy did not correlate with adverse offspring outcomes (major congenital anomalies, spontaneous abortions, ectopic pregnancy, non-live birth) [26,27,28].

Similar data concern the use of GA in pregnancy. Pharmacovigilance analyses on large-size branded databases report that the risk of congenital anomalies overlapped that of the general population [29]. As a matter of fact, while studies investigating the use of IFN and GA during pregnancy were conducted on a wide population, available research on the other molecules belonging to the class of DMTs has been performed on smaller groups.

To the best of our knowledge, few studies analyzed the effect of the maternal use of DMF during the first trimester on the offspring’s adverse outcome, and a correlation was not found [30,31].

As suggested by British Guidelines, fingolimod should be discontinued two months before conception due to reports of malformations and abortions in treated women [32]. However, recent knowledge reports a risk rate of abortions and malformations in MS women similar to that of the general population [33,34,35].

Teriflunomide is currently contraindicated when planning a pregnancy due to a proven teratogenicity in animal research and controversial abortion rates in human studies [35,36].

In pregnancies exposed to natalizumab, no adverse fetal outcome emerged; however, there is the need to assess newborns’ risk of hematological alterations [35,37]. It is crucial to consider that the cessation of natalizumab may result in severe MS reactivation, highlighting the necessity of understanding the right time of drug discontinuation [37]. To date, women with high-activity disease and a high risk of relapse are recommended to continue natalizumab until the beginning of the third trimester and then resume 8–12 weeks after delivery, possibly spacing out infusions during pregnancy [32]. Finally, available knowledge on Ocrelizumab is still very scarce: discontinuation with washout before planning conception is suggested [38]. Overall, the main key points of pharmacotherapy during pregnancy are: washout requirement or timing of discontinuation; evaluation of relapse’s risk after discontinuation.

GA, IFN and DMF do not require washout before pregnancy due to several reasons: a short half-life (DMF), an inability to cross the placental barrier and no evidence of damage to the fetus (GA, IFN) [32,35]. On the other hand, a washout period is strictly recommended for fingolimod (2 months), ocrelizumab (12 months) and teriflunomide (2 years or a complete accelerated elimination procedure) [32]. Although the first indication is to discontinue medications during pregnancy, IFN and GA may be continued during pregnancy, as well as natalizumab, given the significant risk of disease reactivation [32,37]. Indeed, this medication is usually given to patients with highly active disease.

Similarly, there is a high relapse rate after the discontinuation of fingolimod, so, in cases of unexpected pregnancy, the guidelines point to the need for close monitoring or continued treatment in specific cases (e.g., with a history of frequent reactivations) [32,33]. For all these reasons, there are MS pregnancy registers specifically tracking patients who take or have taken medications at the time of conception.

Pregnancy represents a peculiar phase in the life of women with MS. It seems to be linked with positive effects on the course of the disease, it being associated with a decrease in the risk of MS relapses, especially during the third trimester of pregnancy [39]. This effect is probably mediated by a process of immunomodulation on the maternal immune system, induced by sex hormones such as estrogens, which are responsible for a switch in T helper cells from Th1 to Th2, with the production of anti-inflammatory cytokines rather than pro-inflammatory ones [40]. In fact, during the third trimester of pregnancy and during the early postpartum period, an increase in IL-12 levels and a reduction in TNF-a production have been observed [41].

This change is fundamental to developing immune tolerance towards the fetus, avoiding a process of rejection [41]. Consequently, higher levels of estrogens during pregnancy are associated with a reduction in disease relapses [42], while the remarkable decrease in the levels of these hormones after delivery is linked with a relapse rebound during the post-partum period (especially during the first three months) [39,43,44].

It is interesting to notice that the rebound in relapses during the post-partum period is not only represented by a clinical worsening but it is also confirmed by MRI scans, with an increase in the number of T2 hyperintense lesions after the delivery compared to scans performed during pregnancy [45,46].

Thus, MS, as an immune-mediated disorder, could impact fetus development, and, considering the need for and the possibility of pharmacological treatment during pregnancy, the possible influence of medication on developmental trajectories represents a topic of great interest.

The opportunity of providing mothers affected by chronic conditions such as MS with answers concerning the developmental outcome of their children is a key question. However, in order to provide reliable responses, studies with a rigorous methodology are evidently needed.

In this overview, we will summarize and discuss the current literature on the possible impact of maternal MS and the offspring behavioral profile.

## 2. Materials and Methods

A review of the current literature regarding the influence of maternal Multiple Sclerosis on offspring cognitive and behavioral development was carried out in order to overview the available data on the topic.

A study was conducted on Pubmed, Medline and Google Scholar, considering empirical studies and reviews exclusively in the English language. Two authors carried out the research independently.

Table 2 includes the studies that analyze the association between parental Multiple Sclerosis and offspring’s developmental, behavioral and emotional outcomes.

## 3. Results

From a total of 150 articles analyzed that were published during the period 2002–2022, 7 were finally included as specifically focused on neurodevelopmental outcomes in the children of mothers affected by Multiple Sclerosis. Overall, the pool of selected articles involved: 1773 individuals with MS (1464 women; 309 males; mean age of 45.04 yrs) and 1758 offspring (845 females; 785 males; 128 of non-specified gender; mean age of 11.02 yrs). Four of these studies were conducted in Europe (Greece, Italy, Denmark and UK) [47,48,49,50], whilst the others were carried out in Canada and in the USA [51,52,53].

Among the seven studies selected, six did not report an association between maternal MS and the risk of NDDs in offspring [47,48,50,51,52,53] (Table 3). Regarding the possible association between pharmacological treatment for MS during pregnancy and NDDs in children, only one of the selected studies specifically investigated this issue, reporting a significant association [48]. Furthermore, four of the designated studies analyzed the influence of maternal co-occurring psychiatric conditions on offspring’s developmental trajectory, finding an increased risk of NDDs [49,50,51,52]. Notably, the only research reporting a significant relation between maternal MS and the risk of NDDs in offspring [49] described that this risk is even increased when mothers present comorbid psychiatric conditions.

## 4. Discussion

In order to investigate what we know as of now concerning the possible impact of maternal MS on offspring cognitive and behavioral development, we attempted to answer three main questions on the basis of the available literature.

### 4.1. Is Maternal MS Associated with an Increased Risk of Neurodevelopmental Disorders in Offspring?

Most of the studies do not report a significant increase in Neurodevelopmental Disorders (NDDs) risk in the children of women affected by MS [47,48,51,52,53], regardless of whether they are raised in the presence of a concomitant parental psychiatric disorder [49,51,52] (Table 2).

In particular, among parents affected by MS—in comparison to caregivers not affected by chronic illnesses—the presence of a comorbid psychiatric disorder running in families (maternal depression) has been associated with increased child emotional and behavioral problems [49,50].

In addition to this, the maternal MS-related impairment and the illness duration have been related to child developmental vulnerability [40,44], underlining the possible effect of a maternal chronic condition on family functioning and the emotional profile [51,54].

However, even if maternal MS does not seem to be associated with an increased risk of NDDs in offspring, it should be taken into account that adverse developmental trajectory may arise later in age. In fact, most of the studies were conducted on preschool-aged samples [51,52,53], and few studies were conducted in later childhood or adolescence [48,49].

### 4.2. Are the Available Studies on the Topic Methodologically Well Conducted?

The way that studies have been carried out does not allow for conclusions to be drawn. As a matter of fact, even if most of the studies included a large sample size (ranging from 92 to 800 women), several are the methodological limits that characterize these works.

First of all, to the best of our knowledge, none of the studies considered in this manuscript provide a longitudinal neuropsychiatric evaluation of children, but all of them include retrospective data collection (health registries and databases) [47,48,51,52].

Secondly, standardized tools—performed by clinicians—are rarely employed [48,49] in order to measure child development and evaluate behavior, but authors mainly use questionnaires or reports fulfilled by teachers or parents [47,51,52,53]. This modality of acquiring data, relying on parental or teacher perception, does not provide an objective picture of child behavior performed by an expert examiner.

Furthermore, most of the studies are not specifically focused on child development (which includes a standardized assessment of the cognitive profile, adaptive functioning and verbal skills) or on specific behavioral aspects (such as internalizing, externalizing disorders), but these studies more broadly investigate vulnerability issues and developmental milestones through parental or teacher questionnaires [47,49,51,52,53] or even only anthropometric parameters and pediatric developmental abnormalities [53].

As a matter of fact, a reliable and comprehensive child developmental evaluation should include the administration of standardized scales which specifically assess developmental or intellectual quotient (such as Griffith III, Wechsler Scales, Leiter-R and Raven Progressive Matrices), in association with a measure of adaptive skills (such as Vineland Scale or Adaptive Behavior Assessment Scale (ABAS)).

Moreover, behavioral issues should be investigated in different contexts (home, school) through standardized instruments, in addition to the objective behavioral examination performed by the clinician and focused on the aspects that most impair the child (i.e., hyperactivity, anxiousness, isolation, inattention, emotional dysregulation).

Even if Carta and colleagues’ works included a standardized child assessment using valid instruments (ADOS-2, Conners’ Parent Rating Scale, WISC-IV, Leiter-R, Raven Progressive Matrices), however, offspring evaluation was not longitudinally performed. In particular, the children of mothers affected by MS did not primarily undergo a neuropsychiatric evaluation for the purpose of the study, but all MS and control mothers were administered screening questionnaires to ascertain the presence of NDDs in their offspring. In fact, only cases defined as suspected were specifically evaluated for the presence of Autism Spectrum Disorder (ASD), Attention Deficit and Hyperactivity Disorder (ADHD) and Specific Learning Disability (SLD), without showing an increased risk of these disorders [48].

The study of Diareme et al. [49] employed standardized instruments for measuring problematic behavior (Achenbach’s Child Behavior Checklist, CBCL) finding that—in contrast with other studies—more emotional and behavioral problems (internalizing, externalizing) were reported within children exposed to maternal MS in comparison to children of parents without chronic diseases. The employment of these tools may have easily captured such behavioral difficulties in comparison to other unspecific and unvalidated instruments.

### 4.3. Is Maternal Treatment for MS during Pregnancy Associated with an Increased Risk of Neurodevelopmental Disorders in Offspring?

A controversial issue is represented by the possible effect of maternal immune treatment during pregnancy (treatment during pregnancy, discontinued treatment before conception, never treatment) on offspring development (motor and language milestones, cognitive skills, social abilities).

It is essential to consider that drug treatment with DMTs in pregnancy is usually not recommended, so fertile women with MS treated with DMTs received specific guidance regarding contraception [32]. On the other hand, the teratogenic effect has not been confirmed for any medication through studies in humans but rather only in animal models [35]. Although guidelines indicate the discontinuation of DMTs therapy prior to conception, particularly with some molecules, unexpected pregnancies are accidentally exposed. However, only a few studies specifically address the risk of developing NDDs in children of mothers with MS treated with DMTs.

First of all, most of the studies, both animal [30,55,56] and human [26,27,28,35,57,58], investigate the adverse pregnancy outcome (spontaneous abortion, ectopic pregnancy, non-live birth, congenital malformation and, in particular, congenital cardiac defect) rather than the child cognitive and behavioral profile [48,59].

Secondly, since pharmacological clinical trials on pregnant women are not allowed due to ethical issues, the main available information—concerning pregnancy adverse outcomes—refers to retrospective studies or is based on data obtained from accidental assumption during the first trimester of pregnancy [28,60].

Among the few studies specifically evaluating developmental issues, Carta et al. [48] found a weak association between maternal treatment in pregnancy and NDDs in their children. However, the main limits of the study were represented by the lack of homogeneity of the maternal immune treatment (natalizumab, azathioprine and beta-interferon) and the sample size. In fact, the treatment group was constituted by only 13 women (38% of all cases), and treatment information was not longitudinally collected.

Overall, it is not possible to draw conclusions specifically regarding the maternal pharmacological treatment in pregnancy and the effect on children’s cognitive and behavioral development.

Everything mentioned highlights implications for further longitudinal studies, which should: provide direct observation and evaluation of child cognitive and behavioral skills, performed by expert clinicians through the use of standardized instruments at early stages of development until adolescence; be focused on the risk on specific neurodevelopmental disorders; deeply analyze the role of possible maternal confounding factors not strictly related to MS that may negatively impact child neurodevelopment (disentanglement between maternal factors such as comorbid psychiatric disorders, exposure to toxic agents, infections, habits during pregnancy such as smoking, obesity) and exposure to maternal immune treatment; include different age groups of children (from preschool to young adult age) in order to delineate a reliable developmental trajectory and not a static clinical picture which concerns only a phase of development (preschool or school age).

In the context of maternal pathological conditions, to be aware of the possible impact of parental disorders on offspring’s developmental trajectory (depicting the child clinical profile from the first years of life until at least adolescence) represents a key health issue for both parents and clinicians.

## 5. Conclusions

What we know as of now concerning the risk of NDDs in the offspring of mothers affected by MS failed to reach unquestionable conclusions.

Maternal MS appears not to be associated with emotional and behavioral problems (broadly identified as emotional vulnerability), as evaluated through retrospective studies, as far as caregivers and teachers are concerned. However, a specific cognitive and behavioral phenotype has not been delineated yet. Thus, it is not possible to affirm that NDDs risk (i.e., ASD, ADHD, SLD) is not raised within children exposed to the maternal condition of MS. The possible impact of maternal pharmacological treatment in pregnancy on offspring’s cognitive and behavioral outcomes remains an unanswered question in both animal and human studies.

Finally, future research should specifically investigate the possible impact of paternal MS as an autoimmune disease on the children neuropsychological profile.

## Figures and Tables

**Table 1:** Summary of the main knowledge (risk of adverse pregnancy outcome and recommendation) on the use of disease-modifying treatments (DMTs) during pregnancy (interferons (IFN), glatiramer acetate (GA), dimethyl fumarate (DMF), fingolimod, teriflunomide, natalizumab, ocrelizumab).

Medication	Timing of Exposure (Trimester of Pregnancy)	Offspring’s Outcomes	Current Recommendation
IFN	I-II-III	no increased risk of: major congenital anomalies, spontaneous abortions, ectopic pregnancy, non-live birth.	-continue until conception -in the case of unplanned pregnancy: no evidence to consider termination
GA	I-II	no increased risk of: major congenital anomalies, spontaneous abortions, ectopic pregnancy, non-live birth	safe to continue until conception and throughout pregnancy
DMF	I	no increased risk of: inborn defects, premature births, spontaneous abortions	not recommended
fingolimod	I-II-III	no increased risk of: major congenital anomalies	stop at least 2 months prior to conception (washout) and eventually switch to other DMT
teriflunomide	I	no increased risk of: major congenital anomalies, spontaneous abortions, ectopic pregnancy, non-live birth	stop the treatment and use an accelerated elimination procedure
natalizumab	I-II	no increased risk of: major congenital anomalies, spontaneous abortions; haematological adverse event must be better analyzed.	consider treating in pregnancy; last dose: 34 weeks
ocrelizumab	I	no increased risk of: spontaneous abortions or congenital anomalies	stop at least 12 months prior to conception (washout)

**Table 2:** Summary of studies measuring offspring’s cognitive/behavioral outcomes within parents with Multiple Sclerosis.

Year	Authors	Country	Study Design	Parent Sample	Offspring Sample	Control Group	Outcome	Offspring’s Evaluation Tools	Main Findings	Limits	Strengths
2006	Diareme et al.	Greece	Case-control	N = 101 (46 M; 55 F)	N = 56 (31 M; 25 F: MA 9.9 yrs)	N = 128 PARENTS (64 M; 64 F) N = 64 CHILDREN (32 M; 32 F; MA 10.3 yrs)	-Child emotional and behavioral problems -Parental depression -Family disfunction	-CBCL (<11 yrs) -YSR (>11 yrs) -FAD	-Association between maternal MS and offspring’s emotional and behavioral problems -Stronger association in the presence of maternal depression and family dysfunction -MS mothers are more frequently depressed	-Use of teacher, parental, child report -Lack of clinical objective development evaluation	-Control group -Standardized instruments
2015	Razaz et. al.	Canada	Population-based retrospective cohort (health database)	N = 153 (23 M; 130 F)	N = 53 (MA 5.7 yrs)	N = 876 PARENTS without MS and respective OFFSPRING (MA 5.7 yrs)	Child development at 5 years of age	Teacher Report: EDI	-No association between maternal MS and development vulnerability in offspring -Association between maternal psychiatric comorbidity and parental MS duration and development vulnerability in offspring	-Use of teacher report -Lack of clinical objective development evaluation -Retrospective info	-Large sample size -Control group
2016	Bogosian et al.	London, UK	Longitudinal Study (baseline, 6 months distance)	N = 56 MS (mean age: 45.96 yrs), N = 40 partners without MS (mean age: 47.2 yrs)	N = 75 (age range 12–19 yrs)	NO	Internalizing and externalizing problems in offspring	Strength and Difficulties Questionnaire	-Association between parental psychiatric comorbidities and offspring behavioral problems -No association between the severity and type of parental MS and offspring difficulties	-No control group -Lack of clinical objective development evaluation -small sample size	-Longitudinal study
2016	Razaz et. al.	Canada	Population-based retrospective cohort study (health database)	N = 783 (240 M; 543 F)	N = 783 tot. (380 M; 403 F; MA 5.6 yrs).	N = 2988 PARENTS (2211 F; 777 M) N = 2988 CHILDREN (1450 F; 1538 M; MA 5.7 yrs)	Child development at 5 years of age	Teacher Report: EDI	-No association between maternal MS and offspring’s developmental vulnerability -Association between maternal psychiatric comorbidity and offspring’s developmental vulnerability	-Use of teacher report -Lack of clinical objective development evaluation	-Large sample size -Control group
2018	Andersen et al.	Copenhagen (Denmark)	Cohort- and register-based study	N = 382 F	N = 382 (MA 11 yrs) −48.5% M −51.5% F	N = 68.177 PARENTS (F) N = 6.177 CHILDREN (50.3% M 49.7% F MA 11 yrs)	Offspring mental health status at age 11	SDQ (Questionnaire)	-No association between maternal MS and offspring mental status at 11 yrs of age	-Use of teacher, parental, child questionnaires -Register-based study -Lack of clinical development evaluation	-Large sample size -Control group
2021	Carta et al.	Sassary, Italy	Retrospective Observational Study	N = 206 F	N = 361(167 M; 194 F; MA 22.9 yrs) 13 received immune treatment in pregnancy	NO	NDDs	-screening questionnaire -standardized evaluation on a selected sample: WISC-IV, Leiter-R, CPM, SPM, ADOS-2, CPRS.	-No association between maternal MS and NDDs -Weak association between MS treatment during pregnancy and NDDs	-Retrospective study (clinical records) -Enrollment bias	-Control group -Large sample size
2021	Mahlanza et al.	Boston (USA)	Prospective multicenter cohort study	N = 92 F	N = 48 (22 M; 26 F; 0–12 months)	NO	Anthropometric measures (weight, length, head circumference) developmental outcome checklist	-Telephone interviews -Medical records	-No pediatric developmental difficulties -Larger head circumference measurements in children of MS mothers	-No control group -Lack of clinical development evaluation -Focused on anthropometric outcome	-Prospective study -Longitudinal analysis of offspring’s growth

Legend: CBCL: Child Behavior Checklist; CPM: Colored Progressive Matrices; CPRS: Conners Parent Rating Scale; EDI: Early Development Instrument; F: females; FAD: Family Assessment Device; M: males; MA: mean age expressed in years if not otherwise specified; MS: Multiple Sclerosis; NDDs: Neurodevelopmental Disorders; SDQ: Strengths and Difficulties Questionnaire; SPM: Standard Progressive Matrices; WISC-IV: Wechsler Intelligence Scale for Children; YSR: Youth Self Report.

**Table 3:** Main topics addressed by selected studies investigating the relationship between maternal MS and neurodevelopmental outcome in offspring: risk of NDDs, maternal pharmacological treatment for MS and co-occurring maternal psychiatric conditions.

Study	Maternal MS Risk for NDDs	Maternal MS Treatment and NDDs	Maternal MS, Psychiatric Comorbidities and Risk for NDDs	Quality of Methodology
Diareme et al. 2006	Yes	−	Yes	+ + +
Razaz et al. 2015	No	−	Yes	+ +
Bogosian et al. 2016	No	−	Yes	+ +
Razaz et al. 2016	No	−	Yes	+ +
Andersen et al. 2018	No	−	−	+ +
Carta et al. 2021	No	Yes	−	+
Mahlanza et al. 2021	No	−	−	+

Legend: Presence/Absence of association between maternal condition and risk of NDD: Yes/No; Not examined: −. Quality of methodology: Good: + + +; Moderate: + +; Low: +; MS: Multiple Sclerosis; NDDs: Neurodevelopmental Disorders.

## Data Availability

The data presented in this paper are contained within the article.
